# Solvent-Free Catalytic Synthesis of Ethyl Butyrate Using Immobilized Lipase Based on Hydrophobically Functionalized Dendritic Fibrous Nano-Silica

**DOI:** 10.3390/foods14244272

**Published:** 2025-12-11

**Authors:** Mengqi Wang, Yi Zhang, Yunqi Gao, Huanyu Zheng, Mingming Zheng

**Affiliations:** 1 College of Food Science, Northeast Agricultural University, Harbin 150030, China; wangmengqi0624@163.com; 2 Oil Crops Research Institute, Chinese Academy of Agricultural Sciences, Hubei Key Laboratory of Lipid Chemistry and Nutrition, Key Laboratory of Oilseeds Processing, Ministry of Agriculture, Wuhan 430062, China

**Keywords:** lipase immobilization, dendritic fibrous nano-silica, solvent-free esterification, ethyl butyrate

## Abstract

Ethyl butyrate is a typical flavor ester with pineapple-banana scents, but the poor yield from natural fruits limits its feasibility in food and fragrance industries. In this study, dendritic fibrous nano-silica (DFNS) was hydrophobically modified with octyl groups (DFNS-C_8_) to immobilize *Candida antarctica* lipase B (CALB) for solvent-free esterification of ethyl butyrate. The immobilized lipase CALB@DFNS-C_8_, with the enzyme loading of 354.6 mg/g and the enzyme activity of 0.064 U/mg protein, achieved 96.0% ethyl butyrate conversion under the optimum reaction conditions where the molar ratio of butyric acid to ethanol was 1:3, with a reaction temperature and time of 40 °C and 4 h. Under the solvent-free catalytic reactions, CALB@DFNS-C_8_ presented the maximum catalytic efficiency of 35.1 mmol/g/h and retained 89% initial activity after ten reuse cycles. In addition, the immobilized lipase can efficiently catalyze the synthesis of various flavor esters (such as butyl acetate, hexyl acetate, butyl butyrate, etc.) and exhibits excellent thermostability and solvent tolerance. A molecular docking simulation reveals that the hydrophobic cavity around the catalytic triad stabilizes the acyl intermediate and ensures the precise orientation of both acid and alcohol substrates. This work provides new insights into the sustainable production of flavor esters using highly active and recyclable immobilized lipases through rational carrier hydrophobization and structural confinement design.

## 1. Introduction

Flavor esters can exhibit pleasant fragrances such as pears, apples, bananas and pineapples, which are widely used as core ingredients in juices, beverages and baked products [1,2]. In addition, the antioxidant, antibacterial and anti-inflammatory effects highlight the broad applicability of flavor esters in cosmetics, pharmaceuticals and healthcare products [3,4]. For instance, ethyl butyrate is a typical short-chain ester with a strong sweet fruit aroma, similar to pineapple or other tropical fruits [5,6]. Due to its moderate polarity and high volatility, ethyl butyrate can significantly improve the aroma strength and recognition of fruit juice and baked products [7]. Ethyl butyrate is also an efficient oil-phase solvent in pharmaceutical formulations that enhances the solubility and sustained release of hydrophobic drugs [8].

Most esters have naturally existed in fruits and plants, but the poor content, low extraction yield and high processing cost largely limit the production of natural flavor esters [9,10]. In addition, chemical synthesis requires strong acid (such as concentrated sulfuric acid or p-toluenesulfonic acid) to catalyze the high-temperature reflux reactions, causing potential equipment corrosion, high energy consumption and side reactions [11,12]. In contrast, enzymatic esterification via certain lipases can catalyze the reaction between carboxylic acids and alcohols, allowing flavor esters to efficiently and selectively synthesize in a mild and sustainable way [13]. However, the free lipase is prone to conformational changes under organic solvents or high substrate concentration conditions, resulting in a decrease in activity. In addition, the difficulty in recollection and reuse of free lipase makes the enzymatic reactions far more expensive than chemical synthesis [14,15,16]. Hence, enzyme immobilization has been employed to anchor lipases onto solid supports such as silica nanoparticles, metal–organic frameworks (MOFs), covalent organic frameworks (COFs) through physical adsorption, covalent binding, entrapment and crosslinking, etc. [17,18,19,20]. Particularly, hydrophobic adsorption via hydrophobic interaction can immobilize the lipase throughout the carriers with little change to the enzyme’s structures, maintaining its initial catalytic activity [21]. Furthermore, the hydrophobic surface modification on carriers stimulates lipases to open the hydrophobic ‘lid’ structure and expose the active sites, a phenomenon known as interfacial activation [22,23]. Meanwhile, the hydrophobic microenvironment helps remove the by-product water during enzymatic synthesis of flavor esters, thereafter driving the esterification equilibrium toward ester formation [24]. For instance, Liu et al. [25] used octyl (C_8_) to hydrophobically functionalize the silicon carriers, for *Candida antarctica* lipase B immobilization, which achieved 98.0% of ethyl hexanoate in 30 min. Chen et al. [26] found that *Candida* sp. lipase immobilization on octyl-modified ordered mesoporous silica could maintain 60.1% of the lipase’s initial activity after a 10-cycle reuse. At present, catalytic synthesis of ethyl butyrate is generally involving the use of organic solvents including n-heptane, n-hexane and cyclohexane to improve the solubility of substrates, adjust the system polarity and lower the liquid viscosity for enhanced reaction efficiency [27,28,29,30]. However, the volatility, flammability and toxicity of these solvents pose serious safety and environmental risks, making them unsuitable for the production of food-grade products. Moreover, solvents separation and purification are a time-costing and labor-intensive process, which is associated with solvent residue issues [31,32,33]. Hence, enzymatic synthesis of flavor esters in solvent-free conditions are more favorable for the clean, safe and sustainable demands, especially in food and related fields.

In this study, dendritic fibrous nano-silica was prepared and hydrophobically modified to immobilize lipase CALB, which was used to catalyze the solvent-free synthesis of flavor esters. Immobilization conditions such as OTCS (n-Octyltrichlorosilane) modification amount, pH, enzyme concentration and ratio of carrier to enzyme solution were systematically studied based on the enzyme loading, enzyme activity and ethyl butyrate conversion. In solvent-free reaction conditions, enzymatic synthesis of ethyl butyrate was carried out by investigating the effects of the molar ratio of a substrate, the addition of water and immobilized enzyme, and the reaction temperature. In addition, the catalytic stability, reusability and wide adaptability of immobilized lipase were also evaluated, from the perspective of industrial application.

## 2. Materials and Methods

### 2.1. Materials

Lipases used in this study included *Candida antarctica* lipase B (CALB, freeze-dried powder; Amano Enzyme Inc., Nagoya, Japan), *Candida rugosa* lipase (CRL, freeze-dried powder; Sigma-Aldrich, St. Louis, MO, USA), *Porcine pancreas* lipase (PPL, freeze-dried powder; Sigma-Aldrich, St. Louis, MO, USA), *Thermomyces lanuginosus* lipase (NE-10, freeze-dried powder; Vland Biotech, Qingdao, China) and Novozym 435 (8000 U/g; Novozymes, Beijing, China). Tetraethyl orthosilicate (TEOS, 98%) and n-Octyltrichlorosilane (OTCS, 99%), Cetyltrimethylammonium bromide (CTAB), fluorescein isothiocyanate (FITC) and protein concentration detection kit (BCA) were purchased from Shanghai Yuanye Biotechnology Co., Ltd. (Shanghai, China). Butyric acid and n-hexanol were obtained from Aladdin Biochemical Technology Co., Ltd. (Shanghai, China). Acetic acid, butanol, anhydrous ethanol, isopropanol and urea were purchased from Sinopharm Group Chemical Reagents Co., Ltd. (Shanghai, China). p-Nitrophenyl palmitate (p-NPP) was purchased from Sigma-Aldrich (St. Louis, MO, USA). Unless otherwise specified, all reagents and solvents used in the experiment were analytical grade or chromatographic grade.

### 2.2. Preparation of DFNS-C_8_

The synthesis of dendritic fibrous nano-silica (DFNS) is based on the method of Xie et al. [34]. A total of 16.0 g of CTAB and 9.6 g of urea were dissolved in 480 mL deionized water at room temperature, followed by the addition of 480 mL cyclohexane and 3.68 mL isopropanol. Then, 40 mL of TEOS was slowly added into the mixture for 2 h stirring at room temperature and another 20 h reaction at 70 °C. After cooling at room temperature, the whole contents were filtered using a Büchner funnel, washed with pure ethanol and dried overnight at 60 °C. Finally, the obtained solids were calcined at 550 °C in a muffle furnace for 6 h.

The surface modification of DFNS was based on the method reported by Liu et al. [25]. In brief, 1.0 g of DFNS was dispersed in 10 mL n-hexane containing different amounts of OTCS. The mixture was pretreated by ultrasonic treatment (10 min), subsequently reacted (250 rpm, 25 °C, 2 h) and collected by a Büchner funnel. The collected contents were further washed with pure ethanol at least three times and dried in a 60 °C oven overnight to obtain the white solids, namely DFNS-C_8_.

### 2.3. Immobilization and Enzymatic Properties of Lipase CALB

The immobilization of lipase referred to the method of Liu et al. [35]. First, 0.02–0.12 g free lipase CALB was dissolved in 10 mL phosphate-buffer solution (PBS, 50 mM) to prepare lipase solution, 0.1 g DFNS-C_8_ was added and mixed (220 rpm, 30 °C, 40 min). Then, all the contents were centrifuged (7000× *g*, 15 °C, 15 min) to obtain the supernatant, which was used to determine the amount of residual protein by BCA protein assay. At the same time, the solid was collected and freeze-dried (−60 °C, 10 h) to obtain immobilized lipase CALB@DFNS-C_8_. The immobilized enzyme amount was determined by the following formula:

(1) $$Enzyme\;immobilization\;amount\;\left(mg/g\right)=\frac{(C_{0}-C)V}{m}$$

where *C*_0_ is the concentration of lipase before immobilization (g/L), *C* is the concentration of lipase in the supernatant after immobilization, *V* is the volume of the enzyme solution (mL), and *m* is the quality of the added carrier (g).

Enzyme activity was measured using an ultraviolet/visible spectrophotometer based on the hydrolysis of p-nitrophenyl palmitate (p-NPP) in 50 mM, pH 7.0 phosphate-buffer solution (PBS) [36]. Lipase activity was determined by the following formula [37]:

(2) $$Enzyme\;activity\;(U/g)=\frac{A\times V\times n\times 10^{3}}{\varepsilon_{410}\times t\times m}$$

(3) $$Enzyme\;specific\;activity\;(U/mg\;protein)=\frac{Enzyme\;activity}{Protein\;content}$$

where *A* is the absorbance of p-NPP at 410 nm, *V* is the total volume after dilution (mL), *ε*_410_ is the molar absorption coefficient of p-NPP (M^−1^cm^−1^, 14.298 × 10^3^), *t* is the hydrolysis time (min), *n* is the dilution factor and *m* is the mass of immobilized enzyme (g).

The Catalytic efficiency (CE) value was calculated according to the following formula:

(4) $$Catalytic\;efficiency\;(mmol/(g\cdot h))=\frac{S\times Y}{T\times L}$$

where *S* is substrates amount (mmol), *Y* is the yield, *T* is the time of reaction (h) and *L* is the mass of immobilized enzyme (g).

### 2.4. Enzymatic Esterification of Ethyl Butyrate

Ethyl butyrate is enzymatically produced via the esterification between butyric acid and anhydrous ethanol catalyzed by immobilized enzyme CALB@DFNS-C_8_. Specifically, 0.48 g of butyric acid and 0.25–1.25 g of ethanol with molar ratios ranging from 1:1 to 1:5, followed by the addition of 0.062–0.246 g of ultrapure water and 0.019–0.045 g of lipase for the stirring reaction (300 rpm, 4 h, 30–50 °C). During the enzymatic reaction, 20 μL of reaction solution was sampled at regular intervals and diluted with n-hexane. The collected samples were filtered with 0.22 μm PVDF filter and analyzed by high performance gas chromatography. Finally, the immobilized lipase was collected by centrifugation (5000× *g*, 5 min, 15 °C), washed with n-hexane and dried by nitrogen blowing for subsequent reaction cycles.

The yield of ethyl butyrate in the reaction was analyzed using the method described by Bian et al. [38]. The samples were detected by Agilent 7890A gas chromatograph (Agilent Technologies, Santa Clara, CA, USA) equipped with DB-FFAP fused silica capillary column (30 m × 0.25 mm × 0.25 μm) and FID detector. N_2_ was the carrier gas, and the total gas flow rate was set to 21 mL/min. The sample (1 μL) was injected into a split-flow mode with a split ratio of 20:1. The inlet temperature and detector temperature were set at 275 °C and 250 °C, respectively. The specific temperature program is as follows: the column oven was kept at 80 °C for 1 min, then heated to 170 °C at 10 °C/min and then kept for 5 min.

### 2.5. Lipase Performance Evaluation

Temperature stability: The free lipase CALB and CALB@DFNS-C_8_ were incubated at 100 °C for 3 h to evaluate their thermal stability.

Organic solvent tolerance: The free lipase CALB and CALB@DFNS-C_8_ were immersed in n-hexane, isopropanol, acetonitrile, ethanol and acetic acid for 2 h, and the activity of the enzyme was determined after the solvent was dried by nitrogen gas flow.

Reusability: CALB@DFNS-C_8_ was subjected to repeated experiments. At the end of each experiment, lipase was collected by centrifugation and washed with n-hexane for subsequent reaction cycles.

Details of the characterization of CALB@DFNS-C_8_ are provided in Supplementary Method S1.

### 2.6. Molecular Docking Analysis

Molecular docking simulations were performed using AutoDock Tools 1.5.6 to investigate the binding interactions between lipase (CALB, PDB: 1TCA) and acids and alcohols [38]. The three-dimensional structures of acids and alcohols were retrieved from PubChem. Docking parameters, including energy evaluations and grid box dimensions, were set to encompass the catalytic pocket of the enzyme. The Lamarckian genetic algorithm implemented in AutoDock 4.2 was used to perform docking simulations. The lowest-energy conformation was selected as the representative binding mode, and PyMOL (version 2.4.0) and Ligplot (version 2.3) software were used to visually evaluate the docking results.

### 2.7. Statistical Analysis

Statistical significance was evaluated using one-way Analysis of Variance (ANOVA) with a significance level of *p* < 0.05. Data visualization was performed using OriginPro 2021 software (Origin Lab Corp, Waltham, MA, USA).

## 3. Results and Discussion

### 3.1. Lipase Screening

**Figure 1:**
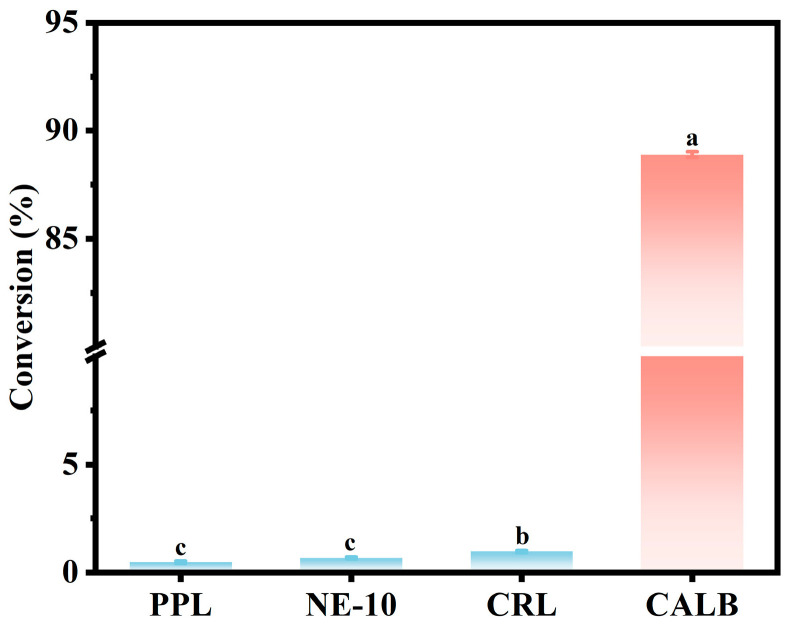
Screening of different lipases based on enzymatic esterification of butyric acid and ethanol. The reaction was carried out in a water bath (40 °C, 300 rpm) for 2 h in an enzyme (3 wt%) with a molar ratio of butyric acid to ethanol of 1:4 and a water content of 10 wt%. A significant difference (*p* < 0.05) between groups is indicated by different lowercase letters.

In this study, different lipases (i.e., PPL, NE-10, CRL and CALB) were investigated based on their catalytic ability to esterify butyric acid and ethanol in a solvent-free system. As shown in Figure 1, the free lipase CALB reached 88.9% of butyric acid conversion in 2 h, while the corresponding conversion rates from lipase PPL, NE-10 and CRL were less than 1%. Notably, Kaur et al. [39] reported that immobilized CRL on magnetic multiwalled carbon nanotubes (MWCNTs) yielded 89.7% ethyl butyrate with n-hexane used as the organic solvent. In contrast, in the solvent-free system in this study, lipase CRL showed limited catalytic performance while lipase CALB maintained higher catalytic efficiency, which could be attributed to its superior regio- and stereo-selectivity as well as strong tolerance and structural stability in acidic conditions [40,41,42]. Thus, lipase CALB was selected for the enzymatic synthesis of ethyl butyrate in this study.

### 3.2. Characterization of DFNS, DFNS-C_8_ and CALB@DFNS-C_8_

**Figure 2:**
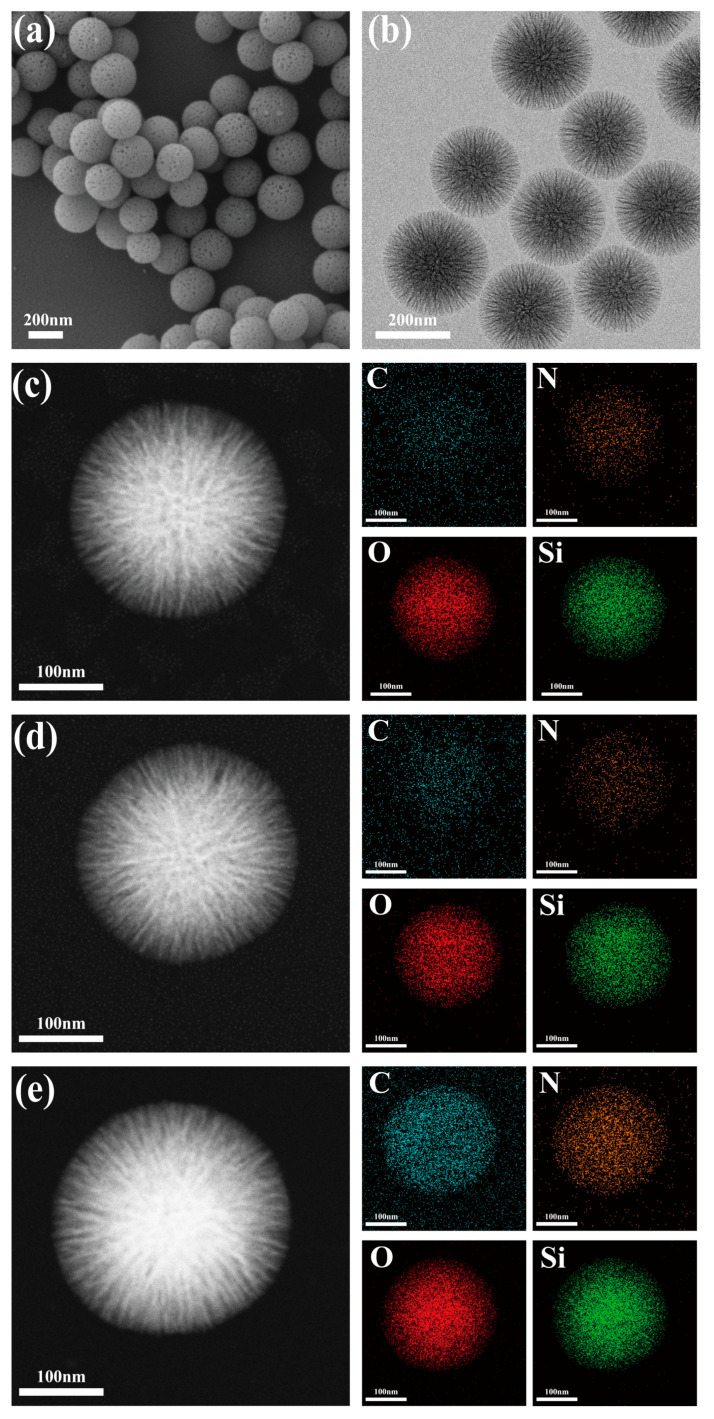
SEM (**a**) and TEM (**b**) images of DFNS. TEM mapping images of DFNS (**c**), DFNS-C_8_ (**d**), CALB@DFNS-C_8_ (**e**).

**Figure 3:**
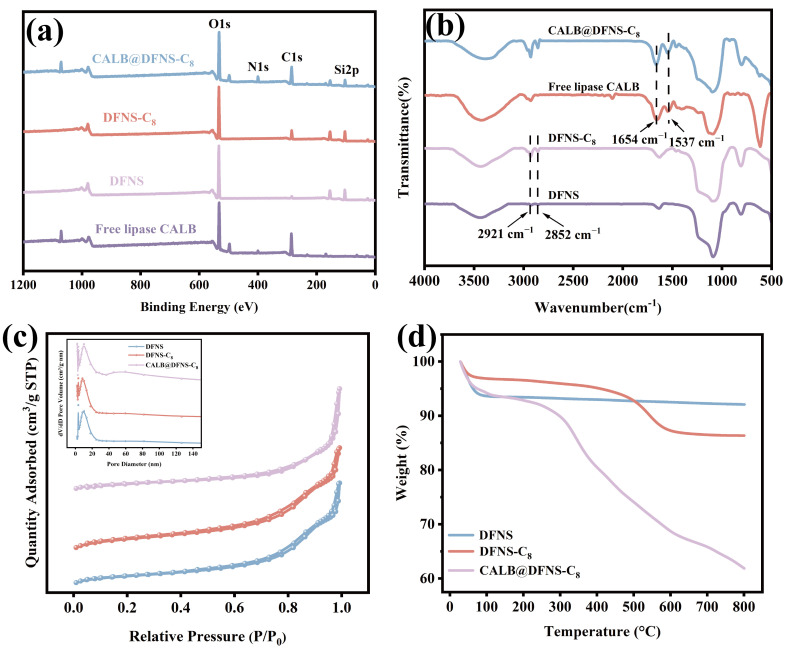
(**a**) XPS images of free lipase CALB, DFNS, DFNS-C_8_ and CALB@DFNS-C_8_. (**b**) FT-IR analysis of DFNS, DFNS-C_8_, free lipase CALB and CALB@DFNS-C_8_. (**c**) N_2_ adsorption–desorption isotherms (adsorption capacity, cm^3^/g STP, relative pressure) and pore size distribution (pore volume, cm^3^/g, pore size, nm) of DFNS, DFNS-C_8_ and CALB@DFNS-C_8_. (**d**) Thermogravimetric analysis of DFNS, DFNS-C_8_ and CALB@DFNS-C_8_.

The prepared dendritic fibrous nano-silica (DFNS) carrier is a complete, uniform mesoporous sphere whose mean diameter is 260 nm (Figure 2a,b). The massive porous structures of DFNS provide sufficient specific surface area for lipase immobilization and subsequent enzyme-substrate contacts [43]. Energy-dispersive X-ray spectroscopy (EDX) shows that the content of C increased from 29.6% to 38.3% after alkyl modification and further increased to 46.6% with lipase CALB immobilization (Table 1; Figure 2c–e). Meanwhile, the alkyl modification and enzyme immobilization had little influence on the morphology of DFNS. XPS results further confirmed the enhancement of the C peak after modification and the emergence of a new N peak after immobilization, which proved the successful alkyl modification on DFNS and lipase immobilization (Figure 3a). FTIR analysis showed that the C-H symmetric and asymmetric stretching vibration peaks of DFNS-C_8_ appeared at 2921 cm^−1^ and 2854 cm^−1^, respectively, while the N-H bending vibration peaks related to the free lipase CALB appeared at 1537 cm^−1^ and 1654 cm^−1^ of CALB@DFNS-C_8_ (Figure 3b). Confocal laser scanning microscopy showed that FITC-labeled CALB was evenly distributed on the carrier DFNS-C_8_ (Figure S1).

The internal structural changes in DFNS with surface modification and subsequent lipase immobilization were characterized based on the specific surface area and pore characteristics. As shown in Figure 3c, DFNS, DFNS-C_8_ and CALB@DFNS-C_8_ all showed type IV isotherms and H1 hysteresis loops, suggesting that the carrier has uniform and well-connected mesoporous channel structure, concentrated pore size distribution and regular pore shape [44]. With alkyl modification, the specific surface area of DFNS decreased from 449.6 m^2^/g to 309.1 m^2^/g, while the mesopores retained their initial size of 8.8 nm (Table 1). The decrease in specific surface area is due to the fact that the grafting of alkyl groups partially covers and occupies the surface of the material, so that nitrogen cannot fully contact the effective surface of mesoporous carrier [45]. Further immobilization of lipase greatly reduced the specific surface area to 48.1 m^2^/g, but the pore size increased to 11.8 nm because the entry of enzyme molecules into the internal cavity can partially block the smaller pores, thereby promoting the proportion of larger pores [46,47]. Thermogravimetric analysis in Figure 3d shows that DFNS had superior thermostability below 800 °C, while DFNS-C_8_ exhibited slight weight loss at 200–600 °C due to the decomposition of octyl chain. The weight loss of CALB@DFNS-C_8_ was about 27% in the same temperature range, corresponding to the thermal decomposition of the enzyme and organic components [48].

### 3.3. Lipase CALB Immobilization

**Figure 4:**
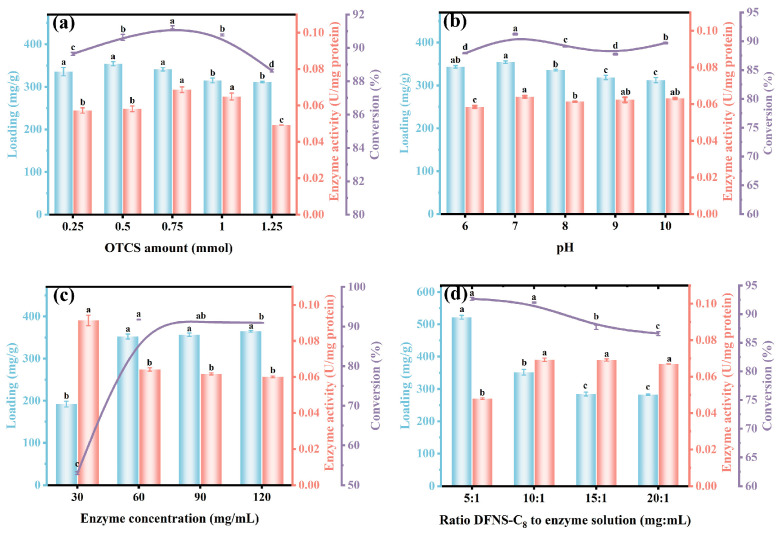
Effects of (**a**) OTCS amount, (**b**) pH of lipase solution, (**c**) enzyme concentration, (**d**) ratio of DFNS-C_8_ to enzyme solution on the loading amount, enzyme activity of CALB@DFNS-C_8_ and ethyl butyrate conversion. The reaction was carried out in a water bath (40 °C, 300 rpm) for 2 h in an enzyme (3 wt%) with a butyric acid and ethanol molar ratio of 1:4. A significant difference (*p* < 0.05) between groups is indicated by different lowercase letters.

Studies have shown that hydrophobic carriers are suitable for immobilization of lipases through interfacial adsorption, because there is a certain interaction between the open conformation of the enzyme and the hydrophobic surface [49]. On this basis, effects of the hydrophobic modification degree on carrier’s hydrophobicity and enzyme immobilization efficiency were studied. With the addition of OTCS increased from 0.25 mmol to 0.75 mmol, the enzyme loading and specific activity were increased from 336.0 mg/g to 341.5 mg/g and from 0.057 U/mg protein to 0.069 U/mg protein, respectively, and were associated with the increased ethyl butyrate conversion from 89.6% to 91.2% (Figure 4a and Figure S1). Further increase in OTCS reversely reduced the enzyme loading, enzyme activity and ethyl butyrate conversion to 315.4 mg/g, 0.065 U/mg protein and 90.8%, respectively. High concentration of OTCS means that more C_8_ molecules were located on the carrier, which inversely hinders the entry of enzymes into the mesopores and results in reduced enzyme immobilization efficiency [25]. Despite the slightly reduced immobilization amount, 0.75 mmol of OTCS induced more hydrophobic, thus enhancing interface activation of CALB@DFNS-C_8_ for higher specific activity and ethyl butyrate conversion [50].

In addition, the effects of OTCS modification on the secondary structures of free CALB and CALB@DFNS-C_8_ were investigated. As shown in Figure S3, increasing the OTCS content of CALB@DFNS-C_8_ from 0 to 0.75 mmol promoted α-helix from 22% to 28% but reduced random coil from 21% to 0. Since higher α-helix content and reduced random coil could improve enzyme rigidity due to increased hydrogen bond, 0.75 mmol of OTCS is concluded to provide the hydrophobic environment needed to maintain the ordered conformation and stability of CALB@DFNS-C_8_. However, increasing the OTCS content to 1.0 mmol caused the proportion of β-sheet to decrease to 28%, accompanied by the conversion rate of ethyl butyrate slightly declining from 91.2% to 90.8%. Hence, 0.75 mmol of OTCS is an optimum value for DFNS hydrophobic modification to maximize the lipase loading and enzyme activity.

The effects of solution pH, enzyme concentration and the ratio of carrier to enzyme solution on lipase CALB immobilization, enzyme activity and catalytic performance were further investigated. As shown in Figure 4b, the maximum immobilization amount (354.6 mg/g), enzyme activity (0.064 U/mg protein) and conversion rate (91.2%) were identified with lipase solution reaching pH 7, whereas minor variations in enzyme loading amount and activity were observed at other pH levels, indicating that the immobilization process on DFNS-C_8_ was relatively insensitive to solution pH under the investigated conditions. Instead, with the increase in enzyme concentration from 30 mg/mL to 60 mg/mL, the immobilization amount increased from 192.1 mg/g to 352.5 mg/g, the enzyme activity decreased (from 0.091 U/mg protein to 0.064 U/mg protein), but the conversion rate increased from 53.0% to 91.7%, relatively (Figure 4c). Although continually increasing the enzyme concentration could slightly increase the lipase loading amount, the enzyme activity and corresponding ethyl butyrate conversion were decreased. The excessive accumulation of lipase CALB on DFNS-C_8_ caused enzyme molecules to be partially obscured or conformationally hindered, thereby reducing the effective activity [51].

Finally, the ratio of DFNS-C_8_ to lipase solution was also observed to affect performance of immobilized lipase. With the ratio increasing from 5:1 to 20:1, the loading amount of lipase CALB, enzyme activity and ethyl butyrate conversion changed from 521.9 mg/g to 282.3 mg/g, 0.048 U/mg protein to 0.067 U/mg protein, and 92.7% to 86.6%, respectively (Figure 4d). Although there were more adsorbed enzymes at 5:1, the accumulation between enzyme molecules would reduce the accessibility of some catalytic sites and cause waste of enzyme resources [52]. Therefore, considering the enzyme utilization rate and catalytic performance, the optimal condition of the ratio of the carrier to enzyme solution was determined to be 10:1.

### 3.4. Optimization of Reaction Conditions

**Figure 5:**
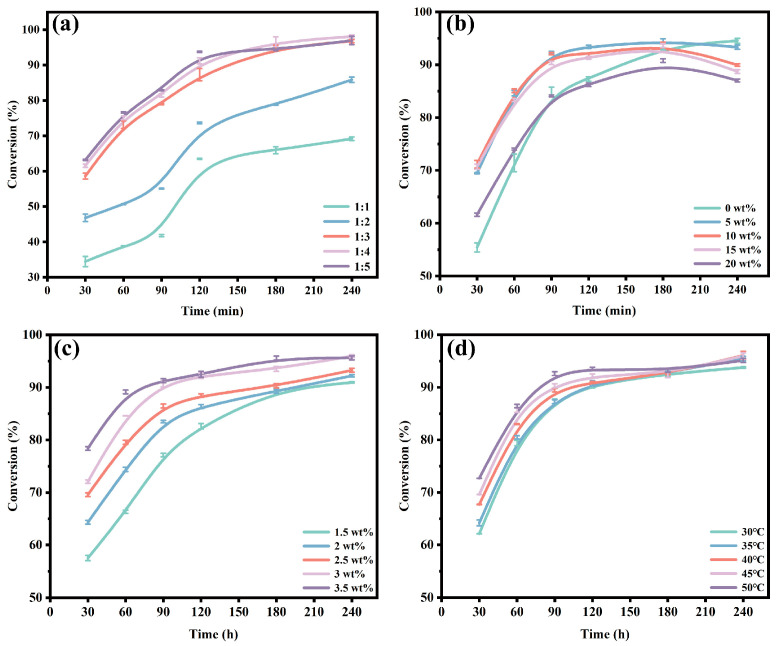
Effects of (**a**) molar ratio of butyric acid to ethanol, (**b**) water addition, (**c**) enzyme addition and (**d**) reaction temperature on the enzymatic synthesis of ethyl butyrate by CALB@DFNS-C_8_.

Based on the above conditions, the optimized immobilized lipase CALB@DFNS-C_8_ was used to catalyze the esterification of butyric acid with ethanol for ethyl butyrate synthesis. As shown in Figure 5a, with the molar ratio of butyric acid to ethanol increased from 1:1 to 1:3, the conversion rate of ethyl butyrate also increased significantly from 69.2% to 96.9%, suggesting more ethanol promoted the contact-catalysis between substrates and immobilized lipase. Continually increasing the molar ratio to 1:5 slightly accelerated the reaction speed but had no further enhancement on the yield of ethyl butyrate. Considering the usage of substrates and corresponding product yield, the substrate molar ratio of 1:3 was selected for the following experiments. The effects of moisture content on enzymatic production were also identified and shown in Figure 5b. A 5 wt% ultrapure water induced 94.4% of butyric acid conversion at 3 h, although it was reduced to 93.3% at 4 h. An appropriate amount of water is conducive to maintaining the activity of lipase, but high water content (10–20 wt%) may cause the hydrolysis of ethyl butyrate [53]. In the initial stage, the conversion rate of the system with 5 wt% water was slightly higher than that of the anhydrous system, and reached the highest 94.4% at 3 h, while the anhydrous system was 93.4% at this time. Thus, the optimal water addition of 5 wt% was determined for the subsequent reactions.

The effects of enzyme addition and reaction temperature on the synthesis of ethyl butyrate catalyzed by CALB@DFNS-C_8_ were also studied. As shown in Figure 5c, as the amount of CALB@DFNS-C_8_ increased from 1.5 wt% to 3 wt%, the conversion rate of ethyl butyrate increased significantly from 91.0% to 96.0%, indicating that more enzyme molecules could provide sufficient catalytic sites to stimulate the substrate conversion rate [54]. Overloaded immobilized lipase had no intensified effects on butyric acid conversion, indicating that 3.5 wt% of CALB@DFNS-C_8_ had exceeded the threshold of enzyme saturation. Similarly, the highest conversion of ethyl butyrate was reached with the reaction temperature being 40 °C (Figure 5d). Increasing the temperature enhanced the catalytic activity of lipase and promoted the movement of substrate molecules and interfacial diffusion [26]. However, the conversion of ethyl butyrate remained constant, as the catalytic activity of the enzyme had reached a relatively stable state in the reaction temperature range of 40–50 °C. In addition, as shown in Figure S4, CALB@DFNS-C_8_ consistently exhibited higher conversion than Novozym435 throughout the reaction period, further confirming its superior catalytic performance.

### 3.5. Catalytic Stability and Applicability Evaluation

**Figure 6:**
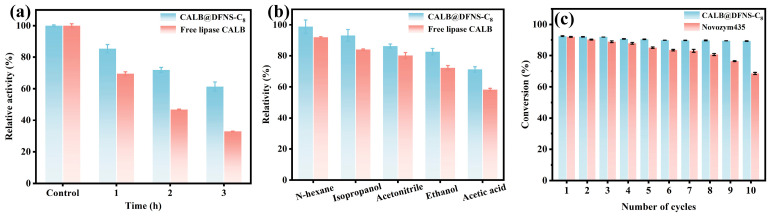
(**a**) Thermostability with 100 °C incubation. (**b**) Organic solvent tolerance of free lipase CALB and CALB@DFNS-C_8_. (**c**) Reusability of CALB@DFNS-C_8_ and Novozym435.

To assess industrial feasibility of enzymatic synthesis of ethyl butyrate, the thermostability, organic solvent tolerance and long-term reusability of CALB@DFNS-C_8_ were assessed. The relative activity of CALB@DFNS-C_8_ gradually decreased to 85.3%, 71.9% and 61.4% with 100 °C incubation for 1 h, 2 h and 3 h, respectively (Figure 6a). In comparison, free lipase CALB was observed to largely lose its catalytic capacity with the same thermal treatment. Enzyme immobilization provides multi-point interaction between the carrier and enzymes to prevent the conformational fluctuations and reduce heat-induced inactivation of lipases [55]. Since the enzymatic synthesis of flavor esters involves organic acids and alcohols, the organic solvent tolerance of immobilized lipase is a key indicator to affect its esterification application. As shown in Figure 6b, compared to free lipase, whose relative activity was drastically reduced after being incubated in n-hexane, isopropanol, acetonitrile, ethanol and acetic acid for 2 h, the corresponding relative activity for CALB@DFNS-C_8_ was maintained at 98.7%, 93.2%, 86.2%, 82.6% and 71.2%, respectively. Especially, the stable activity in high-ethanol environment validated the applicability of CALB@DFNS-C_8_ in catalytic synthesis of flavor esters.

The reusability of immobilized enzymes for the enzymatic synthesis of ethyl butyrate was determined and presented in Figure 6c. Initially, CALB@DFNS-C_8_ could reach 92.7% of butyrate acid conversion, which gradually declined to 89.2% after being consecutively collected and reused for 10 cycles. The biggest bioactivity reduction in immobilized lipase was noticed from the first-round reaction, and was mainly attributed to enzyme leakage from the carrier surface during the collection and separation washing process. During the multiple times of reuse, the performance degradation of immobilized lipase mainly resulted from the mild inactivation, increased mass transfer resistance or trace loss [56]. In contrast, the commercial immobilized lipase Novozym435 showed a more pronounced loss of activity during repeated reuse, with its conversion decreasing to only 68.5% after ten cycles (Figure 6c). Taken together, these results demonstrate that CALB@DFNS-C_8_ not only achieves higher initial catalytic efficiency but also maintains markedly superior operational stability over multiple reuse cycles.

Besides the enzymatic synthesis of ethyl butyrate, CALB@DFNS-C_8_ is also applicable to catalyze the synthesis of acetic acid-based flavor esters (i.e., ethyl acetate, propyl acetate, butyl acetate, pentyl acetate, hexyl acetate) and butyrate acid-based esters (i.e., propyl butyrate, butyl butyrate, pentyl butyrate, hexyl butyrate) with conversion rates of 94.5–98.1% (Table 2). The acetic acid-based esters mainly present sweet pear and banana aromas; butyrate-derived esters pose a more intense tropical fruity aroma, such as pineapple, peach and mango [57,58,59]. These substrates with different carbon-chain lengths all achieved high conversion rates, confirming the high catalytic activity and adaptivity of lipase CALB in catalyzing the production of flavor esters.

**Table 2:** Enzymatic synthesis of various short-chain flavor esters.

Flavor Esters	Acyl Acceptor	Main Flavor Characteristics	Conversion (%)
Ethyl acetate	Ethanol	Sweet, Pear	94. ± 0.4%
Propyl acetate	Propanol	Pear, Banana	95.2 ± 0.1%
Butyl acetate	Butanol	Banana, Apple	95.8 ± 0.4%
Pentyl acetate	Pentanol	Banana	96.5 ± 0.3%
Hexyl acetate	Hexanol	Green apple, Pear	97.9 ± 0.2%
Propyl butyrate	Propanol	Pineapple, Apple	96.0 ± 0.3%
Butyl butyrate	Butanol	Pineapple, Mango	96.1 ± 0.5%
Pentyl butyrate	Pentanol	Pineapple, Peach	97.4 ± 0.2%
Hexyl butyrate	Hexanol	Pineapple, Banana	98.1 ± 0.6%

### 3.6. Molecular Docking Analysis

**Figure 7:**
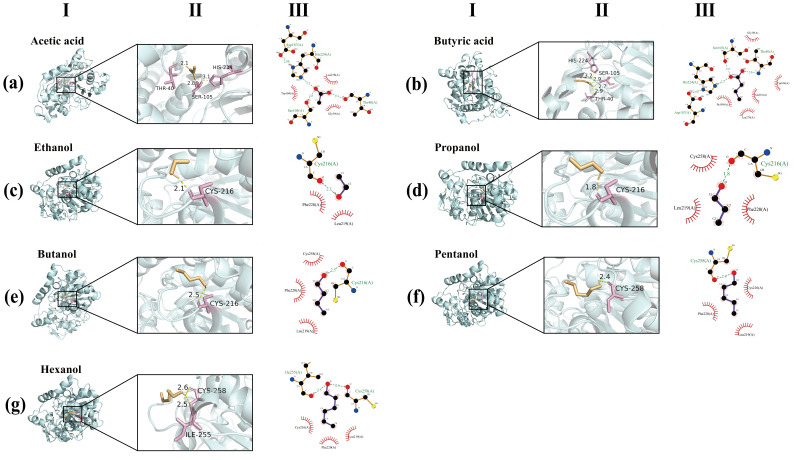
A schematic diagram of the binding process of acetic acid (**a**), butyric acid (**b**), ethanol (**c**), propanol (**d**), butanol (**e**), pentanol (**f**), hexanol (**g**) with lipase CALB. The results include (**I**) and (**II**) 3D molecular docking, as well as the two-dimensional diagram of (**III**) interaction.

In this section, flavor esters synthesis is further explained through the binding mechanisms between lipase CALB and substrates using molecular docking simulation (Figure 7). The molecular docking results revealed that the acids could be stably embedded in the catalytic pocket of CALB, in which the carbonyl oxygen of the acidic substrates is integrated with Ser105 via a hydrogen bond to form the acyl-enzyme intermediates [60]. As an acyl acceptor, the alcohol molecule is located in the peripheral region of the catalytic pocket and assists the subsequent reactions by forming hydrogen bonds with adjacent residues [61,62]. Binding energy results (Table 3; acid: −3.1 to −4.1 kcal/mol; alcohol: −1.6 to −2.5 kcal/mol) indicate that lipase CALB has a stronger affinity for acyl donor (acid), which was consistent with its ping-pong disubstitution mechanism: in the first step, the acidic substrate was subjected to a Ser105 nucleophilic attack under the stability of an oxyanion hole (Thr40, etc.) to form a tetrahedral acyl-enzyme intermediate stabilized by Thr40 hydrogen bond; in the second step, the alcohol, as an acyl acceptor, initiates a nucleophilic attack on the acyl-enzyme intermediate to generate an ester product and regenerate the active site [63]. As the chain length increases, the interaction between the substrate and the hydrophobic residues is enhanced, thereby forming a more stable complex and maintaining a high conversion rate [64]. The disulfide bond of Cys216-Cys258 maintains the structural rigidity of the catalytic pocket and ensures the correct orientation of the substrate [65]. In summary, the high catalytic performance of lipase CALB is mainly attributed to its hydrophobic cavity and conformational rigidity to maintain the precise orientation of the substrate and allow the acid substrate to form a stable acyl-enzyme intermediate at the active center. At the same time, the alcohol substrate completes the deacylation step through the moderate combination of the peripheral hydrophobic pocket, thereby achieving efficient ester bond formation.

**Table 3:** The binding energy of lipase CALB with different substances.

Compounds	Binding Energy (kcal/mol)
Acetic acid	−3.1
Butyric acid	−4.1
Ethanol	−1.6
Propanol	−1.9
Butanol	−2.1
Pentanol	−2.2
Hexanol	−2.5

### 3.7. Comparison of Enzymatic Synthesis of Ethyl Butyrate

The solvent-free enzymatic synthesis of ethyl butyrate using CALB@DFNS-C_8_ was compared with organic solvents-involved studies from the previous literature (Table 4). For example, Monteiro et al. [66] immobilized *Candida antarctica* lipases A and B on magnetic nanoparticles to achieve 99.2% and 97.5% conversion in n-heptane after 6 h, but the whole reactions required a large amount of n-heptane as organic solvent. In the solvent-free system, Paludo et al. [67] achieved 90% ethyl butyrate conversion using 35 wt% Lipozyme TL-IM (*Thermomyces lanuginosus* lipase immobilized on acrylic resin) within 6 h of the reaction period. In contrast, the CALB@DFNS-C_8_ prepared in this work achieved 96.0% conversion of ethyl butyrate in 4 h, leading to a CE of 35.1 mmol·g^−1^·h^−1^ in solvent-free production of flavor esters.

## 4. Conclusions

In this study, a hydrophobically modified DFNS-C_8_ was developed to immobilize *Candida antarctica* lipase B (CALB) for the solvent-free synthesis of ethyl butyrate. Maximum yield of 96% was achieved in 4 h by CALB@DFNS-C_8_, which could maintain 89% of its initial catalytic activity after ten times of reuse. In addition, the immobilized lipase also exhibited superior thermostability, organic solvent tolerance and broad applicability. Molecular docking simulation revealed that the hydrophobic microenvironment of the C_8_ modified carrier allowed the catalytic triad of lipase CALB to stabilize the acyl–enzyme intermediate at low water activity. Overall, this paper provides a green, efficient and recyclable biocatalytic route for flavor ester production based on the rational carrier preparation and hydrophobicity functionalization.

## Figures and Tables

**Table 1:** Elemental contents and text parameters of sample characterization.

Samples	Atomic (%)				BET Analysis		
	C	N	O	Si	Surface Area (m^2^/g)	Pore Volume (cm^3^/g)	Pore Size (nm)
DFNS	29.6	/	52.2	18.2	449.6	1.5	8.8
DFNS-C_8_	38.3	/	45.0	16.7	309.1	1.0	8.8
CALB@DFNS-C_8_	46.6	1.3	37.7	14.4	48.1	0.4	11.8

**Table 4:** Comparison of enzymatic synthesis of ethyl butyrate.

Lipases	Solvents	Reaction Parameters	Conversion (%)	Reusability Cycles (Conversion %)	Catalytic Efficiency (mmol·g^−1^·h^−1^)	References
CRL@Cottoncloth	cyclohexane	1:2.4 ^a^, 25 ^b^, 8 ^c^	91.2	12 (83.0)	1.3	[68]
CRL@PDA-Co-MWCNT	n-heptane	1:3 ^a^, 40 ^b^, 24 ^c^	78.02	6 (49.0)	/	[69]
CRL@MMWCNTs	n-heptane	1:2 ^a^, 35 ^b^, 6 ^c^	89.7	11 (57.0)	/	[39]
RML@CS	n-heptane	1:1 ^a^, 25 ^b^, 6 ^c^	92.0	10 (50.0)	6.1	[70]
Lipozyme TLIM	/	1:1 ^a^, 30 ^b^, 6 ^c^	90.0	10 (90.0)	/	[67]
DMVR46@MWCNTs	n-heptane	0.2:0.15 ^a^, 40 ^b^, 48 ^c^	81.0	15	1.35	[71]
CALB@MNP	n-heptane	1:1 ^a^, 45 ^b^, 6 ^c^	97.5	10 (80)	13.0	[66]
CALA@MNP	n-heptane	1:1 ^a^, 45 ^b^, 6 ^c^	99.2	10 (80)	16.5	[66]
CALB@MNP	n-heptane	1:1 ^a^, 25 ^b^, 8 ^c^	97.8	10 (74)	4.8	[27]
**CALB@DFNS-C_8_**	**/**	**1:3 ^a^, 40 ^b^, 4 ^c^**	**96.0**	**10 (89)**	**35.1**	**This work**

^a^ Butyric acid–alcohol molar ratio; ^b^ Temperature (°C); ^c^ Time (h).

## Data Availability

The original contributions presented in the study are included in the article/Supplementary Materials. Further inquiries can be directed to the corresponding authors.

## Supplementary Materials

The following supporting information can be downloaded at: https://www.mdpi.com/article/10.3390/foods14244272/s1**.** Method S1: Characterization of CALB@DFNS-C_8_. Figure S1: (a) FITC-labeled free lipase CALB. (b) DFNS-C_8_ carrier observed in CLSM bright field. (c) CALB@DFNS-C_8_ is shown by the superposition of green channel and bright field. Figure S2: The water contact angles of unmodified DFNS (a) and 0.25 mmol (b), 0.5 mmol (c), 0.75 mmol (d), 1 mmol (d) and 1.25 mmol octyl modified DFNS-C_8_ were measured. Figure S3: Changes in the secondary structure of free lipase CALB and different octyl modification amounts. Figure S4: Comparison of the catalytic performance of CALB@DFNS-C_8_ and Novozym435. Figure S5: Gas chromatographic diagram of butyric acid and reaction mixture.
