# Supplementary material for: Solvent-Free Catalytic Synthesis of Ethyl Butyrate Using Immobilized Lipase Based on Hydrophobically Functionalized Dendritic Fibrous Nano-Silica
Source: Foods. 2025 Dec 11;14(24):4272. doi: 10.3390/foods14244272 (PMC12732277; doi:10.3390/foods14244272)
Supplement: Supplementary file 1 [file foods-14-04272-s001.zip › foods-4017578-supplementary.pdf]

SUPPORTING INFORMATION

Solvent-free catalytic synthesis of ethyl butyrate using dendritic fibrous nano-silica immobilized lipase

Mengqi Wang [1], Yi Zhang [2,*], Yunqi Gao [2], Huanyu Zheng [1,*] and Mingming Zheng [2]

[1] *College of Food Science, Northeast Agricultural University, Harbin, 150030, China*
[2] *Oil Crops Research Institute, Chinese Academy of Agricultural Sciences, Hubei Key Laboratory of Lipid Chemistry and Nutrition, Key Laboratory of Oilseeds Processing, Ministry of Agriculture, Wuhan 430062, China*
* Correspondence: Corresponding authors.zhangyi07@caas.cn; zhenghuanyu1@163.com

Method S1: Characterization of CALB@DFNS-C_8

The surface morphology and microstructure of DFNS, DFNS-C_8 and CALB@DFNS-C_8 were characterized and analyzed by scanning electron microscopy (SEM, ZEISS GeminiSEM 300, Germany) and transmission electron microscopy (TEM, JEOL JEM-F200, Japan).A computer-controlled nitrogen adsorption analyzer (ASAP 2010, USA) was used to measure the specific surface area, pore size and pore volume of DFNS, DFNS-C_8 and CALB@DFNS-C_8. The characteristic absorption peaks of the samples and the secondary structure changes of lipase CALB were characterized and analyzed by Fourier transform infrared spectroscopy (FT-IR, Bruker, Karlsruhe, Germany). Thermogravimetric analysis (TGA) was performed using a Mettler TGA/DSC1 Thermogravimetric Analyzer, was employed to ascertain the variation in the mass loss of the sample (Switzerland). X-ray photoelectron spectroscopy (XPS; Thermo Scientific ESCALAB Xi +, USA) was performed for further surface chemical analysis. The water contact angle of DFNS-C_8 was measured by contact angle measuring instrument (SINDIN SDC-200S, China). Confocal laser scanning microscopy (LSM 980, ZEISS, Germany) was used to analyze the immobilized distribution of lipase by fluorescence staining imaging.

Figure S1. (a) FITC-labeled free lipase CALB. (b) DFNS-C_8 carrier observed in CLSM bright field. (c) CALB@DFNS-C_8 is shown by the superposition of green channel and bright field.

Figure S2. The water contact angles of unmodified DFNS (a) and 0.25 mmol (b), 0.5 mmol (c), 0.75 mmol (d), 1 mmol (d) and 1.25 mmol octyl modified DFNS-C_8 were measured.

Figure S3. Changes in the secondary structure of free lipase CALB and different octyl modification amounts.

Figure S4. Comparison of the catalytic performance of CALB@DFNS-C_8 and Novozym435.

Figure S5. Gas chromatographic diagram of butyric acid and reaction mixture.

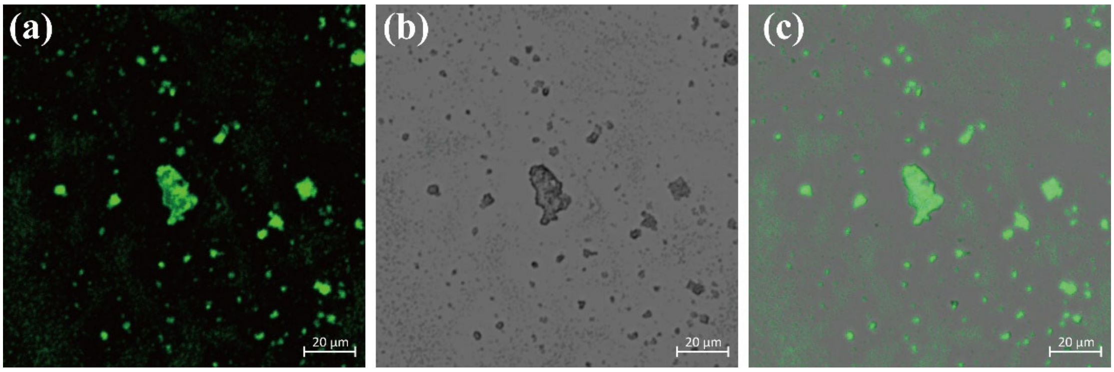

Figure S1.

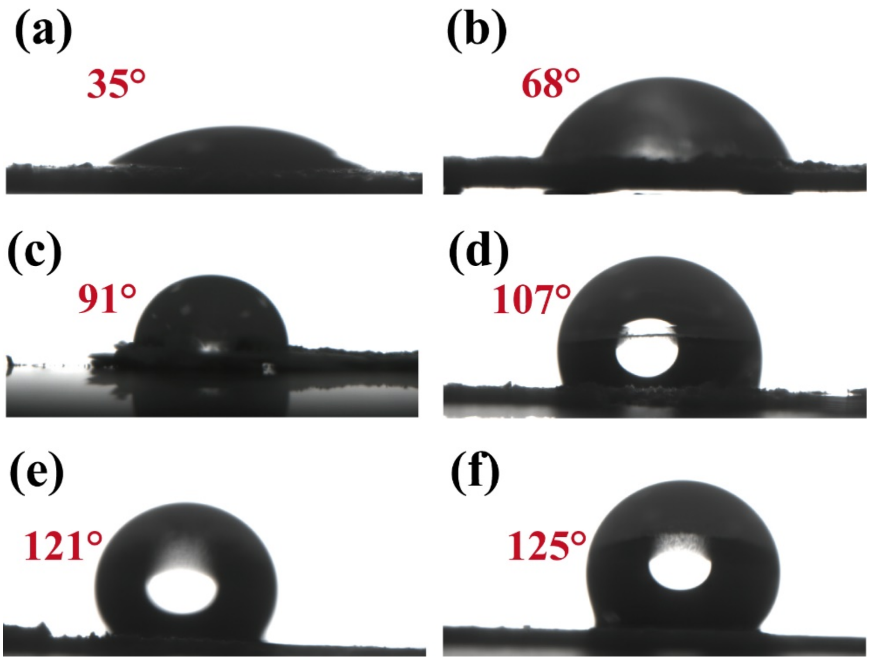

Figure S2.

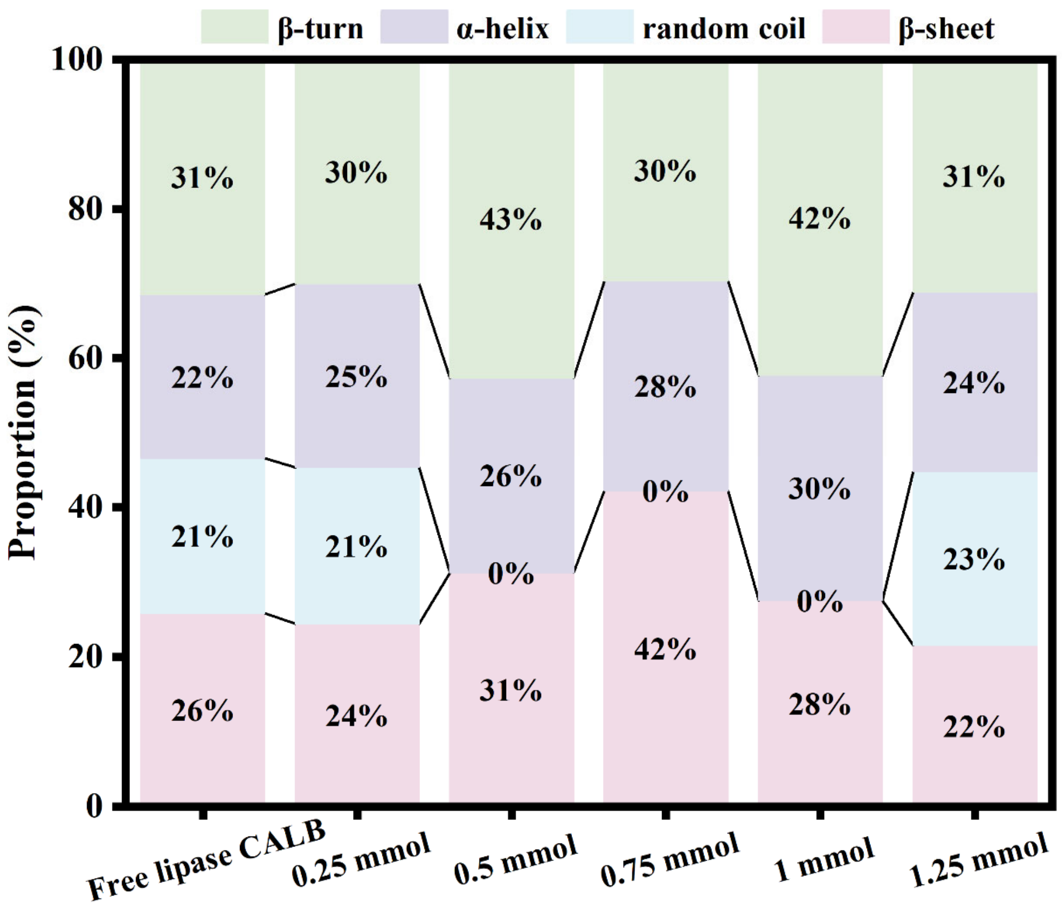
β-turn
α-helix
random coil
β-sheet
Proportion (%)
100
80
60
40
20
0
31%
22%
21%
26%
30%
25%
21%
24%
43%
26%
0%
31%
30%
28%
0%
42%
42%
30%
0%
28%
31%
24%
23%
22%
Free lipase CALB
0.25 mmol
0.5 mmol
0.75 mmol
1 mmol
1.25 mmol

Figure S3.

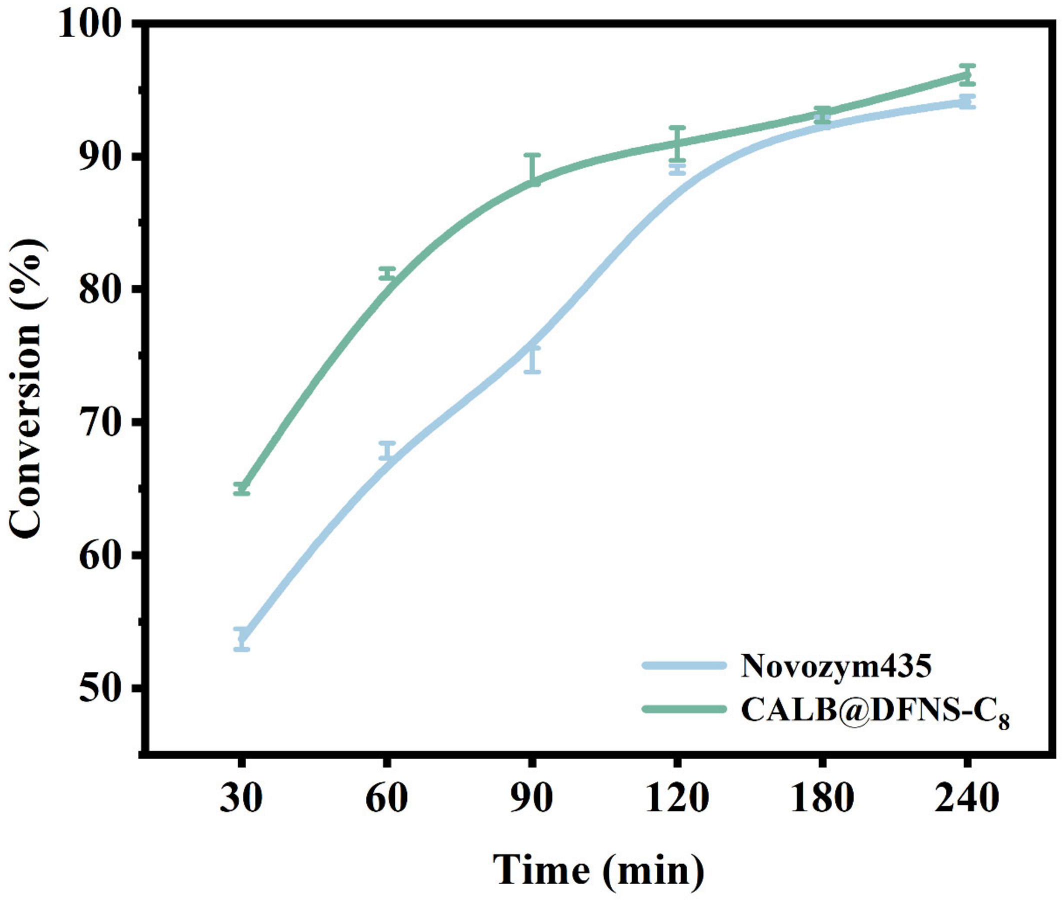
100
90
80
70
60
50
Conversion (%)
30
60
90
120
180
240
Time (min)
Novozym435
CALB@DFNS-C8

Figure S4.

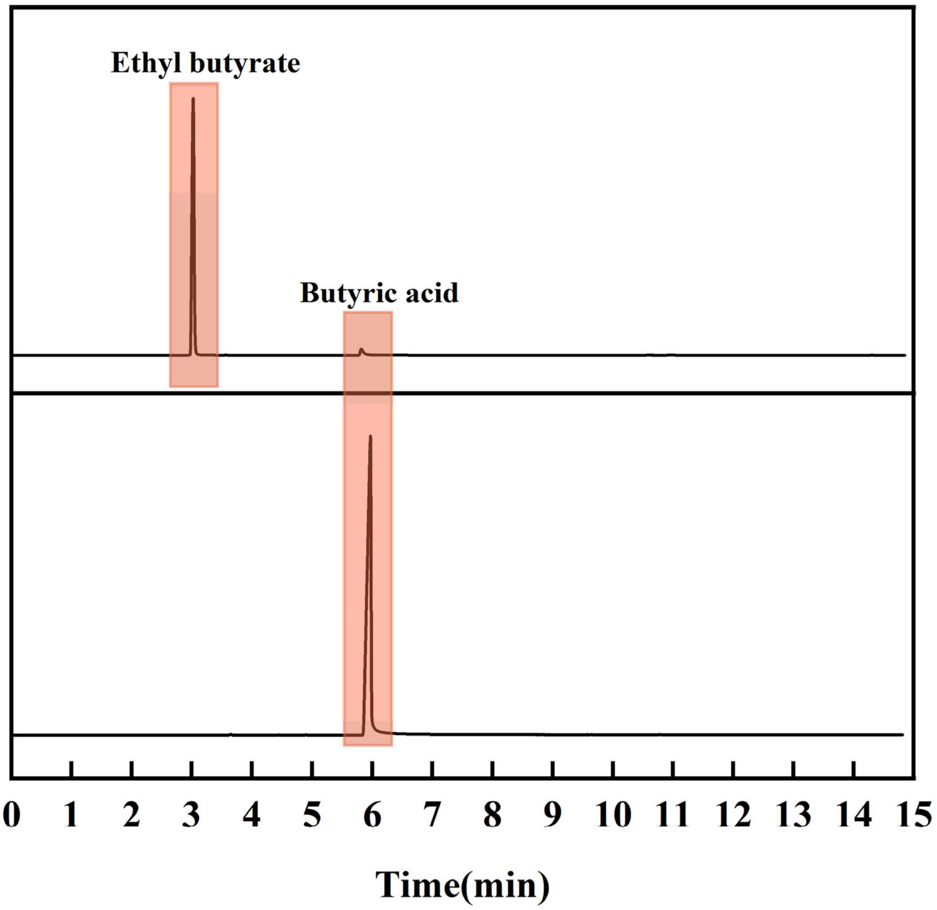
Ethyl butyrate
Butyric acid
0 1 2 3 4 5 6 7 8 9 10 11 12 13 14 15
Time(min)

Figure S5.
